# Dual HDAC/BRD4 Inhibitors Relieves Neuropathic Pain by Attenuating Inflammatory Response in Microglia After Spared Nerve Injury

**DOI:** 10.1007/s13311-022-01243-6

**Published:** 2022-05-02

**Authors:** Vittoria Borgonetti, Elisabetta Meacci, Federica Pierucci, Maria Novella Romanelli, Nicoletta Galeotti

**Affiliations:** 1 Department of Neuroscience, Psychology, Drug Research and Child Health (NEUROFARBA), Section of Pharmacology and Toxicology, University of Florence, Viale G. Pieraccini 6, 50139 Florence, Italy; 2 Department of Experimental and Clinical Biomedical Sciences, “Mario Serio”-Unit of Biochemical Sciences and Molecular Biology, University of Florence, Viale GB Morgagni 50, 50134 Florence, Italy; 3 Department of Neuroscience, Psychology, Drug Research and Child Health (NEUROFARBA), Section of Pharmaceutical and Nutraceutical Sciences, University of Florence, Via Ugo Schiff 6, 50019 Sesto Fiorentino, Italy

**Keywords:** HDAC, BET, Neuropathic pain, Microglia, NF-kB, Cytokines

## Abstract

Despite the effort on developing new treatments, therapy for neuropathic pain is still a clinical challenge and combination therapy regimes of two or more drugs are often needed to improve efficacy. Accumulating evidence shows an altered expression and activity of histone acetylation enzymes in chronic pain conditions and restoration of these aberrant epigenetic modifications promotes pain-relieving activity. Recent studies showed a synergistic activity in neuropathic pain models by combination of histone deacetylases (HDACs) and bromodomain and extra-terminal domain (BET) inhibitors. On these premises, the present study investigated the pharmacological profile of new dual HDAC/BRD4 inhibitors, named SUM52 and SUM35, in the spared nerve injury (SNI) model in mice as innovative strategy to simultaneously inhibit HDACs and BETs. Intranasal administration of SUM52 and SUM35 attenuated thermal and mechanical hypersensitivity in the absence of locomotor side effects. Both dual inhibitors showed a preferential interaction with BRD4-BD2 domain, and SUM52 resulted the most active compound. SUM52 reduced microglia-mediated spinal neuroinflammation in spinal cord sections of SNI mice as showed by reduction of IBA1 immunostaining, inducible nitric oxide synthase (iNOS) expression, p65 nuclear factor-κB (NF-κB) and p38 MAPK over-phosphorylation. A robust decrease of the spinal proinflammatory cytokines content (IL-6, IL-1ß) was also observed after SUM52 treatment. Present results, showing the pain-relieving activity of HDAC/BRD4 dual inhibitors, indicate that the simultaneous modulation of BET and HDAC activity by a single molecule acting as multi-target agent might represent a promise for neuropathic pain relief.

**Supplementary Information:**

The online version contains supplementary material available at 10.1007/s13311-022-01243-6.

## Introduction

The prevalence of chronic neuropathic pain is around 6.9–10% of general population [1] and these estimates are going to rise due to aging population and increased survival to severe chronic pathologies (i.e., cancer, diabetes, etc.). Despite the effort on developing new treatments, therapy for neuropathic pain is still a clinical challenge. Complete pain-relieving activity is usually not achieved and available treatments produced limited efficacy (about 30–50%) in a portion (20–40%) of patients [2]. Furthermore, dose-related side effects can limit tolerability and the higher, more efficacious doses often present intolerable side effects [3, 4]. To address these limitations, combination therapy regimes are often employed, and recent investigations indicate some combinations of two or more drugs as a valuable and safer approach in neuropathic pain states [5, 6].

Accumulating evidence shows an important role of epigenetic enzymes in chronic pain conditions. Altered DNA and histone methylation, histone acetylation, and micro-RNA activity have been described in chronic pain states and restoration of these aberrant epigenetic modifications has been associated with pain-relieving activities [7, 8]. Among these epigenetic events, histone acetylation processing appears to play a key role in chronic pain development and maintenance [9, 10]. Histone acetylation processing involves histone acetylation writers (histone acetyltransferases (HATs)), erasers (histone deacetylases (HDACs)), and readers (bromodomain and extra-terminal proteins (BET)). Histone acetylation is driven by the HATs, which acetylate lysine amino acids on histones promoting gene transcription [11] whereas removal of acetyl groups from histones is catalyzed by HDACs that condense chromatin, and thereby reduce gene transcription [12]. Readers display affinity for specific epigenetic marks on histones to form large multi-protein complexes [13]. Eighteen HDACs have been identified in humans and classified into four groups based on sequence homologies: class I (HDAC 1, 2, 3, and 8); class IIa (HDACs 4, 5, 7, and 9); class IIb includes HDACs 6 and 10; class III (sirtuins 1–7); class IV (HDAC 11) [12]. BET family consists of four proteins: Brd2, Brd3, Brd4, and bromodomain testis-specific protein (BRDT). These proteins contain two N-terminal bromodomains which specifically recognizes and binds acetylated lysine residues on histone tails to promote transcription [14].

Several studies indicated an upregulation of HDACs in neuropathic pain conditions [15–18] and restoration of these aberrant epigenetic modifications by treatment with histone deacetylase inhibitors produced pain-relieving activity in clinical [19] and preclinical studies [20, 21]. Altered spinal activity of BETs has also been described in neuropathic pain models and treatment with BET inhibitors (BETi) ameliorated pain hypersensitivity [22–24]. In addition, recent studies showed a synergistic activity in neuropathic pain models by combination of HDAC and BET inhibitors [25]. An innovative strategy to simultaneously inhibit HDACs and BETs is represented by dual inhibitors, single molecules able to modulate more than one target. Dual HDAC/BET inhibitors have shown improved clinical outcomes as anticancer therapy [26], but their effectiveness in neuropathies has not yet been investigated. Thus, the aim of the present study is to investigate the efficacy of dual HDAC/BET inhibitors in a mouse spared nerve injury model.

## Materials and Methods

### Animals and Ethics Approval

Male CD1 mice (24–26 g, 4 weeks old) from the Harlan Laboratories (Bresso, Italy) were used. Mice were housed under standard conditions as previously described [27]. Experiments were carried out in accordance with international laws and policies (Directive 2010/63/EU of the European parliament and of the council of 22 September 2010 on the protection of animals used for scientific purposes; Guide for the Care and Use of Laboratory Animals, US National Research Council, 2011). Protocols were approved by the Animal Care and Research Ethics Committee of the University of Florence, Italy, under license from the Italian Department of Health (410/2017-PR).

Animal studies are reported in compliance with the animal research: reporting of in vivo experiments (ARRIVE) guidelines [28]. Protocols were designed to minimize the number of animals used and their suffering.

Mice were sacrificed by cervical dislocation for removal of spinal cord for in vitro analyses. The number of animals per experiment was based on a power analysis [29] and calculated by G power software. To determine behavioral parameters, each tested group was comprised of 8 animals.

### Drug Administration Protocol

**Fig. 1:**
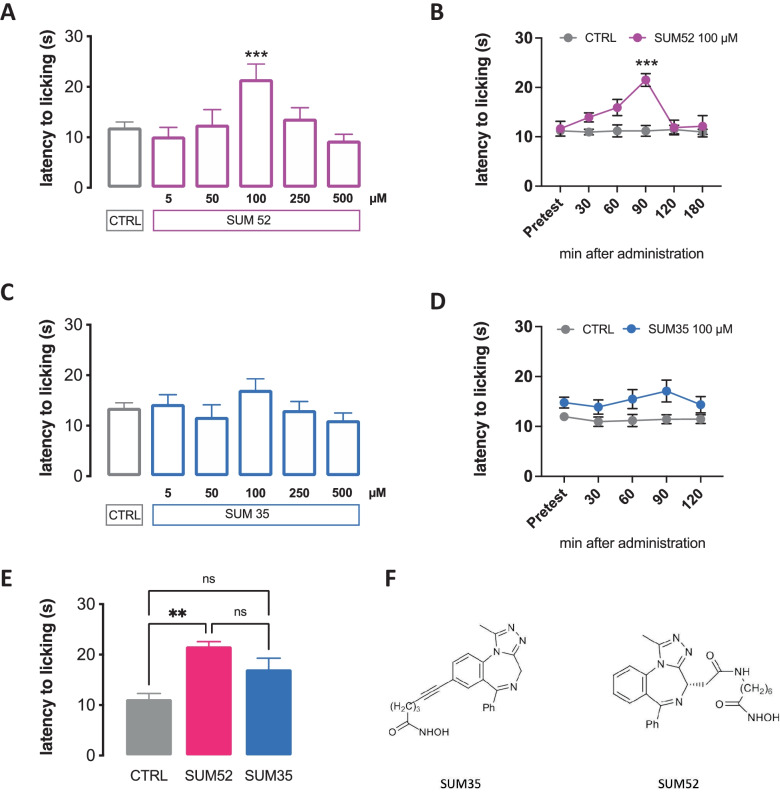
Effect of dual HDAC/BRD4 inhibitors on acute thermal pain. **A** Antinociceptive profile of SUM52 against an acute thermal stimulus (hot plate test). **B** Time course study showing that SUM52 100 μM increased the pain threshold 90 min after the intranasal administration. **C** and **D** SUM35 (5–500 μM) did not produce any significant antinociceptive effect. **E** Normalizing data to the control group, SUM52 100 μM produced a significant analgesic effect compared to SUM35 100 μM treated mice (**C**) (*n* = 8 per group). **F** Chemical structure of SUM35 and SUM52

Mice were randomly assigned to each treatment group. To evaluate the pharmacological profile of SUM35 (N-hydroxy-6-(1-methyl-6-phenyl-4H-benzo[f][1,2,4]triazolo[4,3-a][1,4]diazepin-8-yl)hex-5-ynamide), SUM52 ((S)-N-hydroxy-7-(2-(1-methyl-6-phenyl-4H-benzo[f][1,2,4]triazolo[4,3-a][1,4]diazepin-4-yl)acetamido)heptanamide) (Fig. 1F), synthesized in the laboratory of Prof. Maria Novella Romanelli, iBET762 (molibresib; (4S)-6-(4-Chlorophenyl)-N-ethyl-8-methoxy-1-methyl-4H-[1,2,4]triazolo[4,3,a][1,4] benzodiazepine-4-acetamide, and SAHA (vorinostat; suberoylanilide hydroxamic acid) (Sigma-Aldrich, Italy) were administered 15 min before the tests. Drugs were dissolved in 5% DMSO. Drug concentrations were prepared in such a way that the necessary dose could be administered in a volume of 10 µl per mouse by intranasal (i.n.) administration. The experimental protocol to test the effect of treatments on behavioral and in vitro tests included 3 control groups: untreated, vehicle (5% DMSO), and saline. A representative figure of the experimental protocol in naïve (C) and SNI (F) mice has been inserted in Supplementary Fig. 1.

Treatments were administered on post-surgical day 7 and 14 and spinal cords for in vitro tests were removed at the peak of efficacy of treatments.

### Intranasal (i.n.) Administration

For i.n. administration, mice were slightly anesthetized by 2% isoflurane inhalation and placed in a supine position [30]. A 5 μl aliquot of solution (treatments or vehicle) was slowly dropped alternatively to each nostril with a micropipette tip.

### Spared Nerve Injury (SNI)

Behavioral testing was performed before surgery to establish a baseline for comparison with post-surgical values. Mono-neuropathy was induced by spared nerve injury and this model of pain in mice has been in use for several years [31]. The SNI procedure was performed as previously described [31].

### Nociceptive Behavior

Animals were habituated to the testing environment daily for at least 2 days before baseline testing. To evaluate onset and progression of pain hypersensitivity, neuropathic mice were monitored by measuring nociceptive responses every 30 min for 3 h before surgery or 3, 7, 10, and 14 days after nerve surgery. Experiments were performed on post-surgery day 7 and 14 when the pain hypersensitivity was well established. Each mouse served as its own control, the responses being measured both before and after surgery. All testing was performed with a blind procedure.

#### Mechanical Allodynia

Mechanical allodynia was measured by using Dynamic Plantar Aesthesiometer (Ugo Basile, Bologna, Italy), as described [32]. Nociceptive response for mechanical sensitivity was expressed as mechanical paw withdrawal threshold (PWT) in grams. PWT was quantified by an observer blinded to the treatment.

#### Hargreaves’ Plantar Test

Thermal nociceptive threshold was measured using Hargreaves’ device, as described [33]. Nociceptive response for thermal sensitivity was expressed as thermal paw withdrawal latency in seconds. All determinations were averaged for each animal.

### Locomotor Activity

#### Rotarod Test

The possible alteration of motor performance induced by each treatment was assessed by rotarod test, as previously described [34]. The integrity of motor coordination was assessed as number of falls from the rod in 30 s.

#### Hole-Board Test

The spontaneous locomotor behavior was evaluated by using the hole-board test [35]. Movements of the animal on the plane represent the spontaneous mobility, and the head-dips in the holes by the mice represent the exploratory activity.

### Western Blot Analysis

The lumbar spinal cord was removed 7 and 14 days after surgery and 90 min after the intranasal administration of SUM52, corresponding to the peack of the effect. Samples (*n* = 4 for groups) were homogenized in a homogenization buffer and processed as previously described [36]. Protein samples (40 µg of protein/sample) were separated on 10% SDS-PAGE and transferred onto nitrocellulose membranes (120 min at 100 V) using standard procedures. Membranes were blocked in PBST (PBS containing 0.1% Tween) containing 5% nonfat dry milk for 120 min. Following washings, blots were incubated overnight at 4 °C with specific antibodies against HDAC-1 (1:1000; Santa Cruz Biotechnology Cat# sc-81598, RRID:AB_2118083); Brd4 (1:1000; Santa Cruz Biotechnology, Cat# sc-518021, RRID:AB_2861151); IL-6 (1:1000; Santa Cruz Biotechnology Cat# sc-57315, RRID:AB_2127596); p38 phosphorylated on Thr180/Tyr182 (p-p38, 1:500; Cell Signaling Technology Cat# 4511, RRID:AB_2139682); iNOS (1:250, Santa Cruz Biotechnology Cat# sc-7271, RRID:AB_627810), IBA1 (1:1000; Santa Cruz Biotechnology Cat# sc-32725, RRID:AB_667733), p-NF-kB p65 (1:500; Santa Cruz Biotechnology Cat# sc-136548, RRID:AB_10610391), and IL-1ß (1:1000; Bioss Cat# bs-0812R, RRID:AB_10855142). After being washed with PBS containing 0.1% Tween, the nitrocellulose membrane was incubated with goat anti-rabbit horseradish peroxidase-conjugated secondary antisera (1:10.000) and left for 1 h at room temperature. Blots were then extensively washed and developed using enhanced chemiluminescence detection system (Pierce, Milan, Italy) and signal intensity (pixels/mm^2^) quantified (ImageJ, NIH). The exposition and developing time used was standardized for all the blots. Several reports suggest that commonly used housekeeping proteins are not equally expressed across cell types and experimental conditions and quantification normalization of signal intensity to total protein loading is preferred [37]. For each sample, the signal intensity was normalized to that of total protein stained by Ponceau S and the acquired images were quantified using Image Lab software. Measurements in control samples were assigned a relative value of 100%.

### Immunofluorescence

On postsurgical day 14, animals were perfused transcardially with 4% paraformaldehyde in 0.1 M phosphate buffer (PBS, pH 7.4). After perfusion, lumbar spinal cord was quickly removed and processed as previously described [27, 38]. Four animals per groups were used for this analysis. Lumbar spinal cord transverse sections possessed a thickness of approximately 15 μm. Primary antibody used was IBA1 (1:100; Santa Cruz Biotechnology Cat# sc-32725, RRID:AB_667733). After rinsing in PBS containing 0.01% Triton-X-100, sections were incubated in secondary antibodies labeled with Invitrogen Alexa Fluor 488 (490–525, 1:400; Thermo Fisher Scientific), Invitrogen Alexa Fluor 568 (578–603, 1:400; Thermo Fisher Scientific), Cruz Fluor 594 (592–614, 1:400; Santa Cruz Biotechnology) at room temperature for 2 h. Sections were coverslipped using Vectorshield mounting medium (Vector Laboratories, Burlingame, CA). A Leica DM6000B fluorescence microscope equipped with a DFC350FX digital camera with appropriate excitation and emission filters for each fluorophore was used to acquire representative images. Images were acquired with × 5 to × 40 objectives using a digital camera. The immunofluorescence intensity was calculated by ImageJ (Wayne Rasband, National Institute of Health, USA).

Co-localization of two different labels was measured using EzColocalization plugin (ImageJ). The extent of co-localization was determined by calculating the Mander’s overlap coefficient (MOC) and the Pearson’s correlation coefficient (PCC). MOC measures the percentage of overlap of two signals computationally standardizing size and intensity and is characterized by a range of values between 0 (complete anticolocalization) and 1 (complete colocalization). PCC quantify the correlation between individual fluorophores considering their intensities. PCC is characterized by determined value range: −1, which indicate anticolocalization; +1, which indicates colocalization; 0, which indicates there is no colocalization.

### FRET Bromodomain Binding Assay

The reader assay is a binding assay using AlphaScreen technology FRET assay and was performed by Reaction Biology (www.reactionbiology.com). The biotinylated peptide binding to the reader domain of His-tagged protein is monitored by the singlet oxygen transfer from the Streptavidin-coated donor beads to the AlphaScreen Ni-chelate acceptor beads.

Reaction buffer: 50 mM Hepes, pH7.5, 100 mM NaCl, 0.05% CHAPS, 0.1% BSA, and 1% DMSO (the final DMSO concentration may differ depending on compound stock and test concentrations).

Bromodomain: BRD4-1: RBC Cat# RD-11–140: Recombinant Human Bromodomain containing protein 4, bromodomain 1 (aa 44–170; Genbank Accession # NM_058243), expressed in *E. coli* with N-terminal His-tag. *MW* = 17.8 kDa; BRD4-2: RBC Cat# RD-11–141: Recombinant Human Bromodomain containing protein 4, bromodomain 2 (aa 349–460;Genbank Accession # NM_058243), expressed in *E. coli* with N-terminal His-tag. *MW* = 15.7 kDa. Ligand (C-term-Biotin): Histone H4 peptide (1–21) K5/8/12/16Ac-Biotin. Detection beads: PerkinElmer: Donor beads: Streptavidin-coated donor beads; Acceptor beads: AlphaScreen Ni acceptor beads.

Reaction procedure:

1. Deliver 2.5X BRD in wells of reaction plate except No BRD control wells. Add buffer instead.
2. Deliver compounds in 100% DMSO into the BRD mixture by Acoustic technology (Echo550; nanoliter range). Spin down and pre-incubation for 30 min.
3. Deliver 5X Ligand. Spin and shake.
4. Incubate for 30 min at room temperature with gentle shaking.
5. Deliver 5X donor beads. Spin and shake.
6. Deliver 5X acceptor beads. Spin and shake. Then gentle shaking in the dark for 60 min.
7. Alpha measurement (Ex/Em = 680/520–620 nm) in Enspire.

### HDACs Inhibition Assay

*HDAC assay:* To test the inhibition activity of compounds, the HDAC1 immunoprecipitation (IP) & Activity Assay Kit (Catalog # K342-25; Biovision) was used under the protocol provided by the kit. HCT-116 cell lines were plated in 6 wells and treated with the investigated compounds at 10 µM for 12 h. After incubation, the media was removed, and the cells were washed with PBS (EuroClone) solution (2X). Then, 200 µl/well of lysis buffer with protease inhibitor was added and the cells were scraped and incubated in ice for 30 min. After centrifugation at 10,000 g for 10 min at 4 °C, the supernatant was collected. One hundred micrograms of each extract was incubated with 6 µl of Rabbit HDAC1 antibody and Rabbit IgG and the volume was reached to 500 µl with PBS with protease inhibitors. After incubation at 4 °C overnight a rotary mixer, 25 µl of the protein-A/G bead slurry, previously washed with PBS (2X), was added to the samples and incubated for 1 h at 4 °C. The beads were recovered after 3 washes with 1 ml PBS, by centrifuging at 14,000 g for ~ 10 s and used for HDAC assay.

*HDAC assay preparation*: For each reaction, 168 µl reaction mix containing HDAC assay buffer and HDAC substrate was prepared, added to the sample and to background control tube, and incubated at 37 °C for 2 h. SAHA was used as a positive control. To measure the total HDAC activity, 3 µl of positive control was incubated with 4 µl HDAC Substrate in a final volume of 180 µl with HDAC assay buffer. Then, 20 µl of the developer was added and incubated for 30 min at 37 °C. The samples were centrifuged and 100 µl transferred in a black flat plate. The fluorescence was read at Ex/Em = 380/500 nm with Tecan M1000 plate reader.

### Data and Statistical Analysis

Behavioral test: Results are given as mean ± s.e.m.; eight mice per group were used. One-way and two-way analysis of variance, followed by Tukey and Bonferroni post hoc test, respectively, were used for statistical analysis. Western blotting: Results are given as the mean ± s.e.m. of band intensities. Four mice per treatment group were used and each run was in triplicate. The differences between groups were determined by one-way ANOVA followed by Tukey post hoc test. Immunofluorescence: Immunoreactive areas are mean values (± s.e.m) of three separate experiments. Individual experiments consisted of five tissue sections of each of the 4 animals per group. Differences among mean immunoreactive areas or mean relative areas were statistically analyzed by one-way ANOVA, followed by Tukey post hoc test or Student’s *t* test. For each test, a *P* value less than 0.05 was considered significant. After ANOVA, the post hoc tests were run only if F achieved the necessary level of statistical significance. The computer program GraphPad Prism version 9.0 (GraphPad Software Inc., San Diego, CA, USA) was used in all statistical analyses.

## Results

### Pharmacological Profile of SUM35 and SUM52 in Naïve Mice After Acute Thermal Stimulus

The dual HDAC/BRD4 inhibitors SUM35 and SUM52 were investigated for antinociceptive activities after application of an acute thermal stimulus.

SUM52 showed thermal antinociceptive effects at doses ranging from 5 to 500 µM, 90 min after intranasal (i.n.) administration (Fig. S1B). The dose of 5 µM was ineffective, at 50 µM, there was a trend toward an increase in the pain threshold and the thermal analgesia peaked at 100 µM. Higher doses showed a progressive reduction of efficacy with a bell-shaped trend (Fig. 1A). Time-course studies showed that SUM52 100 µM progressively increased the pain threshold with a peak of antinociceptive activity 90 min after administration. The effect disappeared at 120 min (Fig. 1B). SUM35, in the same range of concentrations did not produce any significant increase of the thermal pain threshold (Figs. 1C, D and S1A). A comparison of the effects produced by both compounds administered at the dose of 100 µM showed the higher efficacy of SUM52 (Fig. 1E). In Fig. 1F, we reported the chemical structure of SUM35 and SUM52.

### SUM52 and SUM35 Attenuation of Nociceptive Behavior in SNI Mice

To complete the antinociceptive profile of the dual inhibitors, their efficacy was investigated in a model of neuropathic pain, the SNI model. Previous results from our laboratory showed that SNI mice develop a persistent thermal allodynia in the injured leg starting from 3 days after surgery that is maintained up to 28 days post-surgery [39]. On the bases of these time-course studies, experiments were conducted on day 7 and 14 after surgery when pain hypersensitivity was well established.

**Fig. 2:**
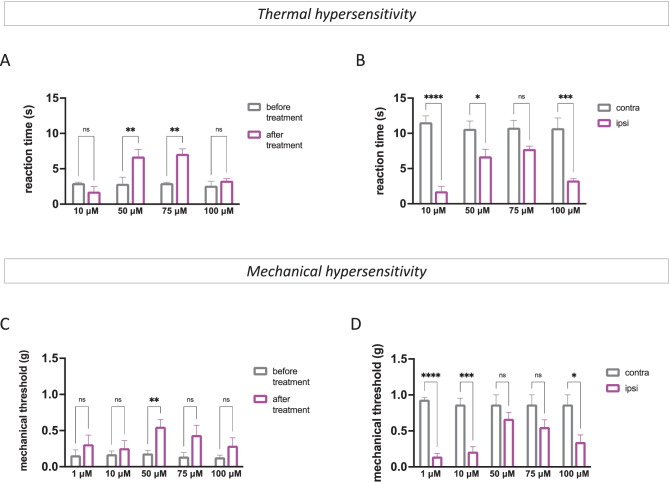
SM52 relieves pain hypersensitivity in SNI mice (*n* = 8 per group). Dose–response curve of SUM52 (10–100 µM) showed thermal (**A**) and mechanical (**C**) antiallodynic activity in the ipsilateral side (ipsi) of SNI mice compared to before treatment values. Comparison of the effect of SUM52 in the contra and ipsilateral side showed the absence of any thermal (**B**) or mechanical (**D**) analgesic effect in the contralateral side

SUM52 (10–100 µM) after 90 min from i.n. administration (Fig. S1E) showed a dose-dependent thermal antiallodynic activity. The dose of 10 µM was ineffective. At 50 and 75 µM attenuated the SNI-induced pain hypersensitivity in the ipsilateral side in comparison with before treatment values. At the dose of 100 µM, the effect was significantly reduced showing a bell-shaped trend (Fig. 2A). Comparison between reaction times recorded in the ipsilateral side (IPSI) and times of the contralateral side (CONTRA), used as internal control, showed thermal threshold values for SUM52 comparable to those recorded in the uninjured leg (Fig. 2B).

In addition to thermal hypersensitivity, as already observed, SNI mice developed an intense and prolonged mechanical allodynia with a time-course similar to that observed for thermal allodynia [40]. SUM52 attenuated SNI-induced mechanical allodynia with a similar profile to that observed for the thermal antiallodynic activity (Figs. 2C, D and S1H).

**Fig. 3:**
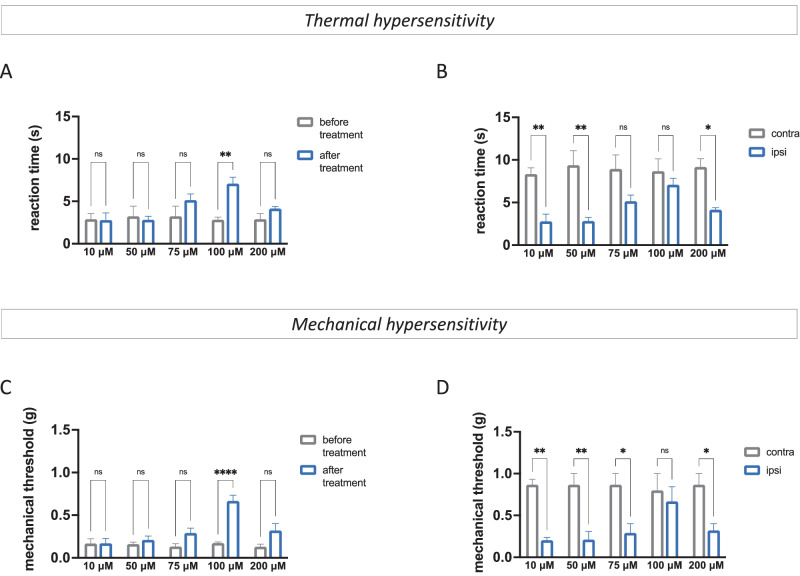
SUM35 attenuated pain hypersensitivity in SNI mice (*n* = 8 per group). Dose–response curve of SUM52 (10–200 µM) showed thermal (**A**) and mechanical (**C**) antiallodynic activity in the ipsilateral side (ipsi) of SNI mice compared to before treatment values. Comparison of the effect of SUM52 in the contra and ipsilateral side showed the absence of any thermal (**B**) or mechanical (**D**) analgesic effect in the contralateral side

Conversely to results from acute pain paradigm, mice treated with SUM35 reduced thermal (Fig. 3A, B) and mechanical (Fig. 3C, D) allodynia in the ipsilateral side in comparison with both ipsilateral side before treatment and contralateral side values, reaching the statistical significance at the dose of 100 µM (Fig. S1D, G). The dose of 200 µM did not produce any significant increase of pain threshold. These results showed that the dual HDAC/BRD4 inhibitors are endowed with antiallodynic activity in the presence of persisting pain rather than analgesic properties in naïve mice.

**Fig. 4:**
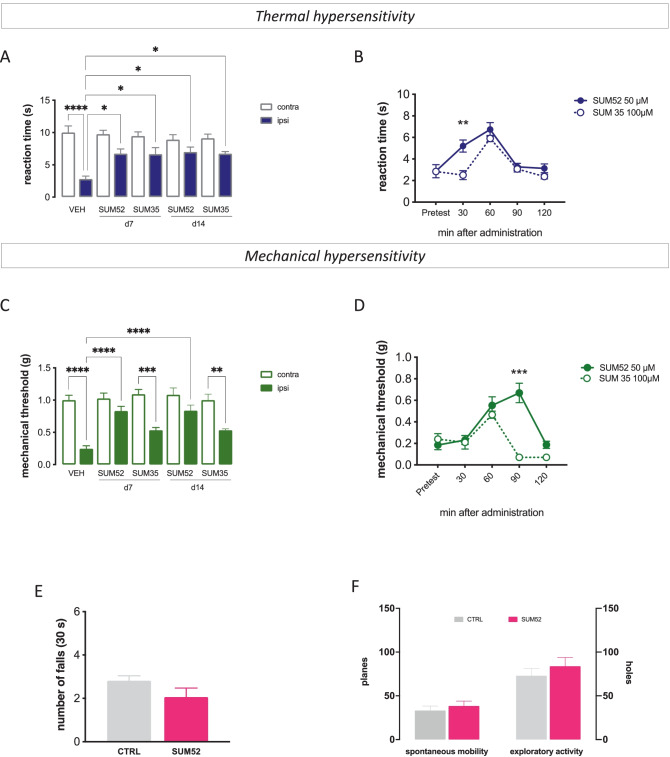
Time-course comparison of the antiallodynic activity of dual inhibitors. Comparison of thermal (**A**) and mechanical (**C**) antiallodynic activity of effective dose of SUM35 and SUM52 normalized to the CTRL group in SNI mice detected on day 7 (d7) and 14 (d14) post-surgery. Comparison of the time-course curves for the highest effective dose of the dual inhibitors against thermal (**B**) and mechanical (**D**) hypersensitivity. Lack of locomotor impairment by SUM52. At the highest effective dose, SUM52 did not alter motor coordination (**E**), spontaneous motility or exploratory activity (**F**) in comparison with vehicle-treated CTRL SNI mice. *n* = 8 per group

By comparing the antiallodynic activity values of the investigated compounds normalized to the control group values, a comparable efficacy in attenuating thermal allodynia was shown by SUM52 (50 µM) and SUM35 (100 µM) (Fig. 4A). Time course studies showed that the peak effect was reached for both molecules 60 min after administration with a more prolonged affect by SUM52 (Fig. 4B). However, SUM52 showed a higher efficacy (Fig. 4C) and more prolonged activity (Fig. 4D) than SUM35 in attenuating mechanical allodynia. Similar results were obtained at day 7 and 14 post-surgery (Fig. 4A, C).

To complete the evaluation of the phenotypical effects produced by dual inhibitors, we tested the most effective molecule on locomotor behavior. No animal showed apparent sedation or motor dysfunction by a single administration of the active dose of SUM52 (50 µM). In addition, no alteration in the motor coordination, evaluated by the rotarod test (Fig. 4E), spontaneous mobility and the exploratory activity (Fig. 4F), evaluated by the hole board test, was detected.

**Fig. 5:**
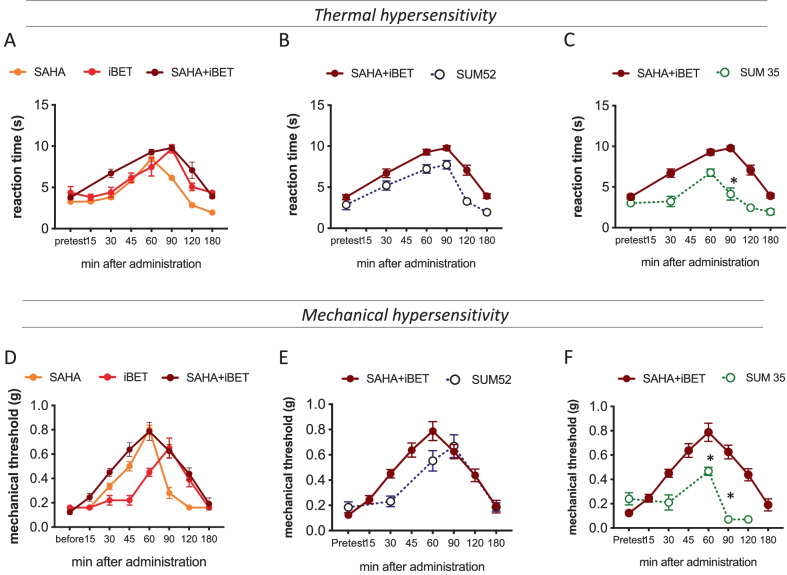
Time-course comparison of the antiallodynic activity of epigenetic modulators. Comparison of thermal (**A**) and mechanical (**D**) antiallodynic activity of SAHA, iBET762 and their combination showing potentiating effects in SNI mice. Comparison between SUM52 and SAHA + iBET762 combination against thermal (**B**) and mechanical (**C**) allodynia showed comparable efficacy between treatments. Comparison between SUM35 and SAHA + iBET762 combination against thermal (**E**) and mechanical (**F**) allodynia showed a lower antiallodynic activity by the dual inhibitor (*n* = 8 per group)

The antiallodynic activity of the investigated dual HADC/BRD4 inhibitors was compared to that produced by combination of the HDAC pan-inhibitor SAHA (vorinostat) and the BRD4 inhibitor iBET762 (molibresib). Co-administration of partially effective doses of the reference drugs (i-BET762 10 µg, SAHA 3 µg) increased both thermal (Fig. 5A) and mechanical (Fig. 5D) pain threshold reaching a complete antiallodynic activity with higher efficacy than full effective doses of each inhibitor (i-BET762 25 µg, SAHA 10 µg) (Fig. 5A, D). SUM52 showed a pharmacological profile that paralleled that of the combined reference drugs against thermal (Fig. 5B) and mechanical (Fig. 5E) allodynia. SUM35 showed a time-course similar to the reference drugs, even though a significantly lower antiallodynic efficacy was recorded (Fig. 5C, F). Consistent with results from acute pain task, no significant effect was produced on the contralateral side at active doses.

### Effects of Dual Inhibitors on HDAC1 and BRD4 Proteins

Previous findings showed a selective overactivation of the HDAC1 isoform with no modification in the expression of HDAC2 [41] and HDAC3 [42] in SNI mice. A prominent role of HDAC1-mediated effects on pain hypersensitivity was also illustrated [18, 42]. Among the BET isoforms, BRD4 is mainly involved in peripheral and central inflammation with a significant role in the pathology of inflammatory diseases [43]. We, thus, decided to focus on HDAC1 and BRD4 isoforms for molecular investigations into the mechanism of action of the dual HADC/BET inhibitors.

**Fig. 6:**
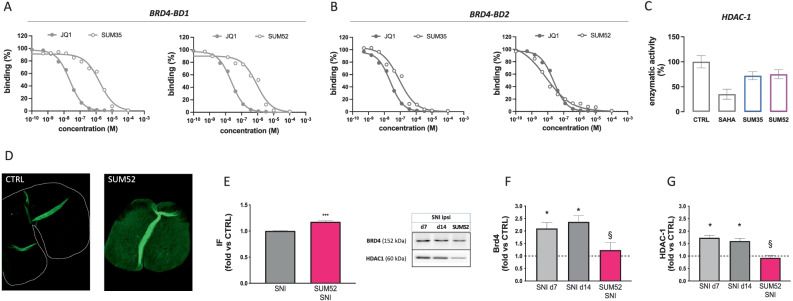
Effect of dual inhibitors on HDAC1 and BRD4 protein. **A** Inhibition curves for SUM35 and SUM52 on BRD4-BD1 in comparison with ( +)-JQ1 (SUM35 IC_50_ = 1722 nM; SUM52 IC_50_ = 758 nM). **B** Inhibition curves for SUM35 and SUM52 on BRD4-BD2 in comparison with ( +)-JQ1 (SUM35 IC_50_ = 118 nM; SUM52 IC_50_ = 10.5 nM). **C** Partial inhibition of HDAC1 enzymatic activity by SUM35 and SUM52 in comparison with SAHA. Microphotographs (**D**) and quantification analysis (**E**) of intrinsic fluorescence of SUM52 detected in spinal cord samples of SNI mice after i.n. delivery compared to vehicle-treated control mice. ***P < 0.001 vs CTRL (**F**) SNI procedure increased the expression of spinal BRD4 in the ipsilateral side on day 7 (d7) and 14 (d14) post-surgery that returned to basal values after SUM52 treatment. **G** Spinal cord samples from SNI mice showed an increased expression of HDAC1 protein in the ipsilateral side (SNI) on d7 and d14 that was completely prevented by SUM52 administration (SUM52 SNI). Dashed lines represent the protein expression value from SNI contra. *P < 0.05, **P < 0.01 vs contra; §*P* < 0.05, §§*P* < 0.01 vs vehicle-treated SNI ipsi (SNI)

The capability to inhibit HDAC1 and BRD4 activity was firstly investigated. BET proteins are characterized by the presence of two tandem N-terminal bromodomains (BD1 and BD2) and a C-terminal “extra-terminal” (ET) domain. The BET bromodomain proteins can bind to two N-ε-acetylated lysine residues on histones and non-histone proteins that are simultaneously recognized by the same bromodomain module [44]. SUM35 and SUM52 were both able to bind BRD4-BD1 (Fig. 6A) and BRD4-BD2 (Fig. 6B) domains with a preferential interaction for BRD4-BD2. Studies on the evaluation of the capability of the SUM35 and SUM52 to inhibit HDAC1 activity showed a partial inhibition by both compounds (Fig. 6C).

SUM52 showed a better pharmacological and biochemical profile than SUM35. Thus, further investigations on the molecular effects were performed on spinal cord tissue from SUM52-treated SNI mice. Following i.n. delivery, SUM52 was able to reach the spinal cord and diffuse into the tissue, as showed by detection (Fig. 6D) and quantification (Fig. 6E) of the intrinsic fluorescence of the molecule in comparison with vehicle-treated mice.

The expression of BRD4 (Fig. 6F) and HDAC1 (Fig. 6G) proteins was increased in lumbar spinal cord samples of SNI mice on day 7 and 14 post-surgery. Treatment with SUM52 restored both epigenetic enzyme levels to control values.

### Reduction by SUM52 of Microglia Activation

**Fig. 7:**
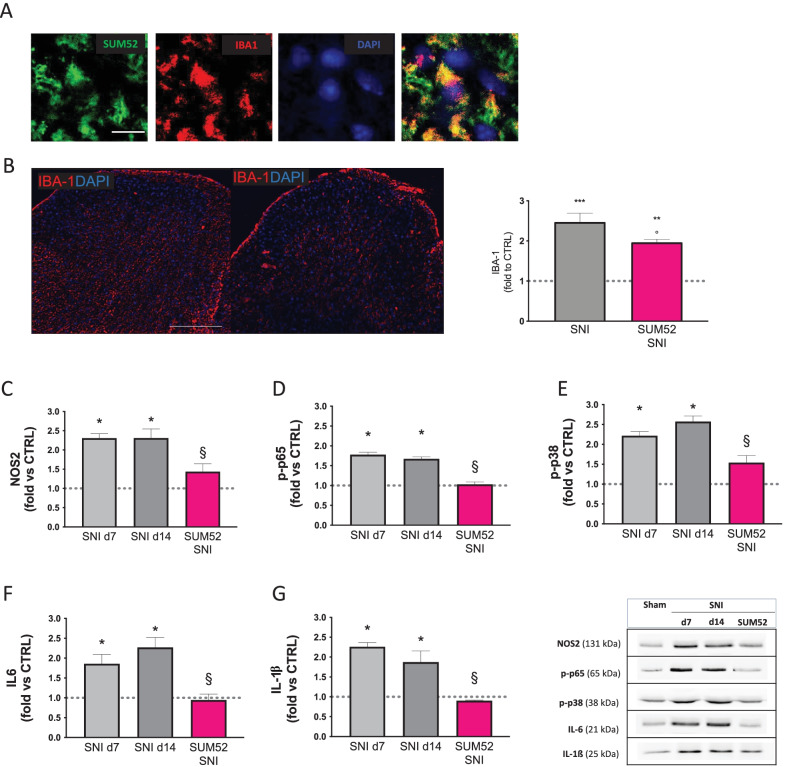
Effect of SUM52 on SNI-induced microglia activation and neuroinflammation. **A** Intrinsic fluorescence of SUM52 was detected in IBA1 positive microglial cells of SNI mice (merged images). Scale bar = 20 µm. **B** Immunofluorescence images of lumbar spinal cord of SNI mice (scale bar 100 µm) with the quantification analysis that showed the reduction of IBA1 immunostaining by SUM52. Dashed lines represent IBA1 immunostaining value from SNI contra. **P < 0.01, *** P < 0.001 vs contra; *P* < 0.05 vs vehicle-treated SNI ipsi (SNI). Spinal cord samples from SNI mice, collected on day 7 (d7) and 14 (d14) post-surgery, showed an increase of the proinflammatory microglia marker iNOS (**C**), increased phosphorylation of the proinflammatory transcription factor p65 (**D**) and p38 MAPK (**E**), increased levels of proinflammatory cytokines IL-6 (**F**) and IL-1ß (**G**) in the ipsilateral side that were reduced up to basal levels by SUM52. Dashed lines represent the protein expression value from SNI contra. *P < 0.05, **P < 0.01 vs contra; §*P* < 0.05, §§*P* < 0.01 vs vehicle-treated SNI ipsi (SNI)

Following nerve damage, a remarkable microgliosis in the spinal cord is usually detected and large evidence demonstrated the contribution of activated microglia in the development of pain hypersensitivity [45]. We evaluated whether SUM52 was able to reduce microglia activation in spinal cord sections of SNI mice. Intrinsic fluorescence of SUM52 was detected in IBA1 positive microglial cells of SNI mice (Fig. 7A) and treatment significantly reduced immunostaining of the microglia marker IBA1 (Fig. 7B). In addition, neuropathic mice exhibited markedly increased expression of NOS2, a marker of proinflammatory microglia in the ipsilateral side. A comparable increase was detected at both day 7 and 14 post-surgery. SUM52 reduced NOS2 overactivation (Fig. 7C) indicating the prevention of microglial activation after nerve injury by dual HDAC/BRD4 inhibition.

### Attenuation of Neuroinflammation by SUM52

The activation of microglia in the brain and spinal cord after 14 days from surgery promoted the release of proinflammatory mediators, leading to neuroinflammation which had been associated to increased nerve excitability and neuropathic pain [46]. We, thus, investigated the effects produced by SUM52 treatment on the expression of proinflammatory mediators in spinal cord samples from the ipsilateral side of SNI mice. The SNI procedure activated the nuclear factor-kB (NF-kB) pathway, a driver of microglia activation. Western blotting experiments showed an increased phosphorylation of the p65 subunit of NF-kB (Fig. 7D) that was robustly reduced by SUM52 up to baseline values. SNI mice also showed a robust over-phosphorylation of MAPK p38 (Fig. 7E) and increased expression of the proinflammatory cytokines IL-6 (Fig. 7F) and IL-1ß (Fig. 7G). Similar results were obtained at day 7 and 14 post-surgery. SUM52 treatment drastically reduced the aberrant expression of the above-mentioned mediators up to control values.

## Discussion

Increasing evidence indicates a significant role in chronic neuropathic pain conditions of epigenetic enzymes involved in histone acetylation modifications [9, 47]. However, neuropathic pain, as many other neurological disorders, is a multifactorial disorder with a complex pathophysiology in which genetic, epigenetic, lifestyle, and individual factors contribute to the disease. Therapeutic agents targeting only one cellular mechanism have not yet been successful. Therefore, an effective therapy may require more complex therapeutic approaches that consider targeting more than one pharmacological target. On these bases, multi-target drugs acting simultaneously on several disease-relevant targets are expected to be a better therapy. In the present study, we provide the first evidence for a pain-relieving activity of dual HDAC/BRD4 inhibitors.

The analgesic activity resulting from HDAC inhibition in chronic pain conditions has been observed in numerous clinical and preclinical studies. More recently, several investigations have showed that BET inhibition attenuated the hypersensitivity to pain in models of central and peripheral neuropathic pain [25]. Combinations of drugs with an epigenetic mechanism of action are mainly studied as anticancer therapy and several preclinical trials showed the synergistic antitumor activity of combined HDAC/BET inhibitors in comparison with single agent therapy [48–50]. A very recent study from our research group reported that the combination of submaximal effective doses of SAHA (an HDAC inhibitor) and i-BET762 (a BRD4 inhibitor) improved pain-relieving activity in a mouse model of neuropathic pain compared with full effective doses of the single agents [51]. These positive results further supported the hypothesis of a better management of neuropathic pain states by a simultaneous inhibition of HDAC and BRD4 enzymes. We, thus, designed, synthesized, and evaluated for pain-relieving activity new dual HDAC/BRD4 inhibitors, labeled SUM35 and SUM52, as an innovative pharmacological intervention for neuropathic pain.

Targeting more than one disease-relevant target with multi-target ligands appears to be superior to a combination therapy. The use of one molecule eliminates the risk of drug interactions between the combined agents and reduces the risk of drug interactions with other medications. There is also a lower risk of adverse effects. Preclinical data suggest strong synergy at reduced doses with BRD4 and HDAC inhibitors, which has the potential to avoid the overlapping toxicities associated with these two drug types [52]. Different pharmacokinetics of individual agents (divergent bioavailability, requirement for different delivery routes, etc.) might hamper the use of several drugs in one medical preparation. In a model of neuropathic pain, the analgesic activity of SAHA and i-BET762 peaked 60 and 90 min after administration, respectively [51], limiting the additive or synergistic interaction when administered simultaneously. Dual inhibitors offer a simplification of dosage regimen with improved compliance by the patients [53].

Dual inhibitors were tested in animal models of acute and persistent neuropathic pain. Both molecules showed a long-lasting pain-relieving activity against thermal and mechanical allodynia in SNI mice with a pharmacological profile comparable to that showed by the combination of SAHA and iBET762. SUM52 showed higher efficacy as pain-relieving agent than SUM35 in persistent pain tests and produced acute thermal antinociception in naïve mice. Conversely, SUM35 did not induce any significant increase in the pain threshold in acute pain models. Moreover, in animals with neuropathy, both SUM52 and SUM35 were more effective in reducing pain symptoms than in naïve animals, highlighting how the role of HDAC and BRD4 is greater in the central nervous system after injury than after acute pain. Thus, a personalized therapy for the use of these molecules in the clinic research could be assumed.

The dual HDAC/BRD4 inhibitory mechanism of action of the investigated molecules was confirmed by in vitro assays. In the SNI model, an overexpression of the HDAC1 isoform has been observed in the ipsilateral side from day 7 to 21 after surgery [18, 42, 54] with no modification in the expression of HDAC2 [41] and HDAC3 [42]. Thus, both compounds were investigated in vitro for interaction with HDAC1 isoform showing a partial inhibitory activity. In addition, a high binding affinity toward BRD4 was detected. Consistent with behavioral results, SUM52 showed a better affinity profile. The dual mechanism was also confirmed in SNI mice. At analgesic doses, SUM52 reduced the spinal overexpression of HDAC1 and BRD4 induced by the peripheral neuropathy. Both molecules showed a prominent effect in modulating the activation of the BRD4-BD2 bromodomain. The preferential interaction with BD2 appears favorable since BD2 selective inhibitors have demonstrated fewer toxicities [55] and were predominantly effective in models of inflammatory and autoimmune disease [56], making them safer and more suitable therapies for neurodegenerative disorders, including neuropathic pain conditions.

Inflammation in the CNS, called neuroinflammation, is triggered by the body to protect the brain from damage [57]. However, excessive or persistent neuroinflammation damages neuronal cells. Emerging evidence suggests that neuroinflammation is a key player in the onset and development of neuropathic pain [58]. Microglia are the main effector cells of neuroinflammation in the central nervous system [59]. Thus, the effect on microglia activation of SUM52, the best performing dual inhibitor in behavioral tests, was investigated. Following i.n. treatment with SUM52, the intrinsic fluorescence of the molecule was detected in SNI spinal cord samples, showing the capability to reach the spinal tissue with a noninvasive delivery route. SUM52 was detected in microglia cells and quantification analysis of spinal cord sections of SNI-treated mice showed a reduction of IBA1 immunostaining, indicating the capability of SUM52 to counteract proinflammatory microglia activation. Microglia in the activation state promote inflammation by the upregulation of iNOS, the activation of NF-κB pathway, and the release of pro-inflammatory cytokines such as IL-1β and IL-6 [60]. SUM52 treatment reduced the overexpression of proinflammatory cytokines, iNOS, and pP38 MAPK and prevented the activation of the NF-κB signaling pathway, showing an attenuation of neuroinflammation induced by the neuropathy. High HDAC expression is an important factor in promoting neuroinflammation. Class I HDACs are highly expressed in microglia [61] and are usually related to the inflammatory response of glial cells [62] by the expression and release of proinflammatory factors (e.g., IL-6, IL-1β, and TNFα) [63] through the activation of the NF-κB signaling pathway. Several HDAC inhibitors have been demonstrated to produce anti-inflammatory activities through a reduction of proinflammatory responses in microglia [64, 65]. Similarly, BET inhibition attenuated inflammatory responses in microglial cell lines [66, 67] mainly by the modulation of NF-kB activation [68]. Among the BET family members, BRD4 seems prominently involved in a pro-inflammatory response by activating transcription of NF-κB and NF-κB-dependent inflammatory genes [43, 69]. Consistent with these observations, SUM52 prevented the activation of the NF-κB signaling pathway with an efficacy similar to that of SAHA and i-BET762 used in combination. In addition, a robust reduction of cytokine and p-P38 levels was induced by SUM52 treatment in comparison to a modest modulation of IL-6 and IL-1ß produced by SAHA and i-BET762 combination. These results show a pain-relieving activity of SUM52 comparable to that produced by a combinatorial regimen. Indeed, a more pronounced attenuation of the pro-inflammatory response was produced by the dual inhibitor highlighting a promising activity toward neuroinflammatory conditions.

We evaluated the efficacy of epigenetic dual inhibitors following i.n. delivery, a simple and non-invasive route of administration. The i.n. route can transport drugs directly to the brain, and it also allows delivery to the spinal cord of macromolecules [70]. I.n. delivery has been previously compared to intrathecal administration and comparable efficacy was obtained [51, 71]. This administration route allows self-medication in patients, and it may result beneficial for drugs acting within the central nervous system.

In the present study, new dual HDAC/BRD4 inhibitors were investigated as pain-relieving agents. These compounds have been designed to assure simultaneous modulation of BET and HDAC activity with a single molecule to avoid potential pharmacokinetic limitations of the combination therapy. Findings show that SUM52, the most promising dual inhibitor investigated, attenuates pain hypersensitivity in the SNI model with an efficacy comparable to the combination of SAHA (HDAC inhibitor) and i-BET762 (BRD4 inhibitor), but with a more efficient attenuation of spinal neuroinflammation. Results are obtained following i.n. administration, a non-invasive delivery system, to increase the potential translation to a clinical use. The favorable pharmacological profile of HDAC/BRD4 dual inhibitors indicates that the development of multi-target agents might represent a promise for neuropathic pain relief.

## Supplementary Information

Below is the link to the electronic supplementary material.

Supplementary file1 (PDF 227 kb). Supplementary Figure S1. Time course of the effect on acute pain of SUM35 (A; 10, 50, 100, 250 and 500 µM) and SUM52 (B 10, 50, 100, 250 and 500 µM) at 30, 60, 90, 120 and 180 min, using the Hot Plate Test in naïve mice. n=8 for each group. C) Experimental protocol of SUM35 and SUM52 intranasal administration. Time course of the effect on persistent pain of SUM35 (D; 10, 50, 100, 250 and 500 µM) and SUM52 (E; 10, 50, 100, 250 and 500 µM) at 30, 60, 90, 120 and 180 min, using the Plantar Test in SNI mice. n=8 for each group. F) Experimental protocol used for SNI mice. Time course of the effect on persistent pain of SUM35 (G; 10, 50, 100, 250 and 500 µM) and SUM52 (H; 10, 50, 100, 250 and 500 µM) at 30, 60, 90, 120 and 180 min, using the Von Frey Test. n=8 for each group

Supplementary file2 (PDF 1224 kb)

Supplementary file3 (PDF 1224 kb)

Supplementary file4 (PDF 1224 kb)

Supplementary file5 (PDF 1224 kb)

Supplementary file6 (PDF 1224 kb)

## References

[1] Van Hecke O, Austin SK, Khan RA, Smith BH, Torrance N (2014). Neuropathic pain in the general population: a systematic review of epidemiological studies. Pain.

[2] Finnerup NB, Attal N, Haroutounian S, McNicol E, Baron R, Dworkin RH (2015). Pharmacotherapy for neuropathic pain in adults: a systematic review and meta-analysis. Lancet Neurol.

[3] Alles SRA, Smith PA (2018). Etiology and pharmacology of neuropathic pain. Pharmacol Rev.

[4] Finnerup NB, Kuner R, Jensen TS (2021). Neuropathic pain: from mechanisms to treatment. Physiol Rev.

[5] Mathieson S, Kasch R, Maher CG, Zambelli Pinto R, McLachlan AJ, Koes BW (2019). Combination Drug therapy for the management of low back pain and sciatica: systematic review and meta-analysis. J Pain.

[6] Chaparro LE, Wiffen PJ, Moore RA, Gilron I (2012). Combination pharmacotherapy for the treatment of neuropathic pain in adults. Cochrane Database Syst Rev.

[7] Descalzi G, Ikegami D, Ushijima T, Nestler EJ, Zachariou V, Narita M (2015). Epigenetic mechanisms of chronic pain. Trends Neurosci.

[8] Polli A, Godderis L, Ghosh M, Ickmans K, Nijs J (2020). Epigenetic and miRNA expression changes in people with pain: a systematic review. J Pain.

[9] Ligon CO, Moloney RD, Greenwood-Van MB (2016). Targeting epigenetic mechanisms for chronic pain: a valid approach for the development of novel therapeutics. J Pharmacol Exp Ther.

[10] Odell DW (2018). Epigenetics of pain mediators. Curr Opin Anaesthesiol.

[11] Marmorstein R, Zhou MM (2014). Writers and readers of histone acetylation: structure, mechanism, and inhibition. Cold Spring Harb Perspect Biol.

[12] Seto E, Yoshida M (2014). Erasers of histone acetylation: the histone deacetylase enzymes. Cold Spring Harb Perspect Biol.

[13] Filippakopoulos P, Picaud S, Mangos M, Keates T, Lambert JP, Barsyte-Lovejoy D (2012). Histone recognition and large-scale structural analysis of the human bromodomain family. Cell.

[14] Filippakopoulos P, Qi J, Picaud S, Shen Y, Smith WB, Fedorov O (2010). Selective inhibition of BET bromodomains. Nature.

[15] Cui SS, Lu R, Zhang H, Wang W, Ke JJ (2016). Suberoylanilide hydroxamic acid prevents downregulation of spinal glutamate transporter-1 and attenuates spinal nerve ligation-induced neuropathic pain behavior. NeuroReport.

[16] Danaher RJ, Zhang L, Donley CJ, Laungani NA, Hui SE, Miller CS (2018). Histone deacetylase inhibitors prevent persistent hypersensitivity in an orofacial neuropathic pain model. Mol Pain.

[17] Denk F, Huang W, Sidders B, Bithell A, Crow M, Grist J (2013). HDAC inhibitors attenuate the development of hypersensitivity in models of neuropathic pain. Pain.

[18] Sanna MD, Guandalini L, Romanelli MN, Galeotti N (2017). The new HDAC1 inhibitor LG325 ameliorates neuropathic pain in a mouse model. Pharmacol Biochem Behav.

[19] Niesvizky R, Ely S, Mark T, Aggarwal S, Gabrilove JL, Wright JJ (2011). Phase 2 trial of the histone deacetylase inhibitor romidepsin for the treatment of refractory multiple myeloma. Cancer.

[20] Vojinovic J, Damjanov N (2011). HDAC Inhibition in rheumatoid arthritis and juvenile idiopathic arthritis. Mol Med.

[21] He XT, Hu XF, Zhu C, Zhou KX, Zhao WJ, Zhang C (2020). Suppression of histone deacetylases by SAHA relieves bone cancer pain in rats via inhibiting activation of glial cells in spinal dorsal horn and dorsal root ganglia. J Neuroinflammation.

[22] Palomés-Borrajo G, Badia J, Navarro X, Penas C. Nerve excitability and neuropathic pain is reduced by bet protein inhibition after spared nerve injury. J Pain [Internet]. 2021;22(12):1617–30. Available from: https://www.sciencedirect.com/science/article/pii/S152659002100242X. 10.1016/j.jpain.2021.05.005. 10.1016/j.jpain.2021.05.005 34157407

[23] Sánchez-Ventura J, Amo-Aparicio J, Navarro X, Penas C. BET protein inhibition regulates cytokine production and promotes neuroprotection after spinal cord injury. J Neuroinflammation [Internet]. 2019;16(1):124. Available from: 10.1186/s12974-019-1511-7. 10.1186/s12974-019-1511-7 PMC6560758 31186006

[24] Borgonetti V, Galeotti N. Combined inhibition of histone deacetylases and BET family proteins as epigenetic therapy for nerve injury-induced neuropathic pain. Pharmacol Res [Internet]. 2021;165:105431. Available from: https://www.sciencedirect.com/science/article/pii/S1043661821000141. 10.1016/j.phrs.2021.105431. 10.1016/j.phrs.2021.105431 33529752

[25] Romanelli MN, Borgonetti V, Galeotti N (2021). Dual BET/HDAC inhibition to relieve neuropathic pain: recent advances, perspectives, and future opportunities. Pharmacol Res.

[26] Zhang S, Chen Y, Tian C, He Y, Tian Z, Wan Y, et al. Dual-target inhibitors based on BRD4: novel therapeutic approaches for cancer. Curr Med Chem. 2021;28(9):1775–95. 10.2174/0929867327666200610174453. 10.2174/0929867327666200610174453 32520674

[27] Sanna MD, Ghelardini C, Galeotti N (2015). Activation of JNK pathway in spinal astrocytes contributes to acute ultra-low-dose morphine thermal hyperalgesia. Pain.

[28] McGrath JC, Lilley E (2015). Implementing guidelines on reporting research using animals (ARRIVE etc.): new requirements for publication in BJP. Br J Pharmacol.

[29] Charan J, Kantharia N (2013). How to calculate sample size in animal studies?. J Pharmacol Pharmacother.

[30] Bortolozzi A, Castãé A, Semakova J, Santana N, Alvarado G, Cortés R (2012). Selective siRNA-mediated suppression of 5-HT1A autoreceptors evokes strong anti-depressant-like effects. Mol Psychiatry.

[31] Bourquin AF, Süveges M, Pertin M, Gilliard N, Sardy S, Davison AC (2006). Assessment and analysis of mechanical allodynia-like behavior induced by spared nerve injury (SNI) in the mouse. Pain.

[32] Sanna MD, Les F, Lopez V, Galeotti N (2019). Lavender (Lavandula angustifolia Mill.) essential oil alleviates neuropathic pain in mice with spared nerve injury. Front Pharmacol.

[33] Hargreaves K, Dubner R, Brown F, Flores C, Joris J (1988). A new and sensitive method for measuring thermal nociception in cutaneous hyperalgesia. Pain.

[34] Sanna MD, Borgonetti V, Galeotti N. μ opioid receptor-triggered notch-1 activation contributes to morphine tolerance: role of neuron–glia communication. Mol Neurobiol. 2019. 10.1007/s12035-019-01706-6 31347026

[35] Borgonetti V, Governa P, Biagi M, Galeotti N (2020). Novel therapeutic approach for the management of mood disorders: in vivo and in vitro effect of a combination of L-theanine, Melissa officinalis L. and Magnolia officinalis Rehder & E.H. Wilson. Nutrients.

[36] Sanna MD, Mello T, Masini E, Galeotti N (2018). Activation of ERK/CREB pathway in noradrenergic neurons contributes to hypernociceptive phenotype in H4 receptor knockout mice after nerve injury. Neuropharmacology.

[37] Mishra M, Tiwari S, Gomes AV (2017). Protein purification and analysis: next generation western blotting techniques. Expert Rev Proteomics.

[38] Borgonetti V, Galeotti N. Fluorescence colocalization analysis of cellular distribution of MOR-1. In: Spampinato SM, editor. Opioid Receptors: Methods and Protocols [Internet]. New York, NY: Springer US; 2021. p. 27–34. Available from: 10.1007/978-1-0716-0884-5_3. 10.1007/978-1-0716-0884-5_3 32975786

[39] Sanna MD, Lucarini L, Durante M, Ghelardini C, Masini E, Galeotti N (2017). Histamine H4 receptor agonist-induced relief from painful peripheral neuropathy is mediated by inhibition of spinal neuroinflammation and oxidative stress. Br J Pharmacol.

[40] Borgonetti V, Governa P, Biagi M, Pellati F, Galeotti N (2020). Zingiber officinale Roscoe rhizome extract alleviates neuropathic pain by inhibiting neuroinflammation in mice. Phytomedicine.

[41] Maiarù M, Morgan OB, Tochiki KK, Hobbiger EJ, Rajani K, Overington DWU (2016). Complex regulation of the regulator of synaptic plasticity histone deacetylase 2 in the rodent dorsal horn after peripheral injury. J Neurochem.

[42] Sanna MD, Galeotti N (2018). The HDAC1/c-JUN complex is essential in the promotion of nerve injury-induced neuropathic pain through JNK signaling. Eur J Pharmacol.

[43] Huang M, Zeng S, Zou Y, Shi M, Qiu Q, Xiao Y (2017). The suppression of bromodomain and extra-terminal domain inhibits vascular inflammation by blocking NF-κB and MAPK activation. Br J Pharmacol.

[44] Sanchez R, Meslamani J, Zhou MM (2014). The bromodomain: from epigenome reader to druggable target. Biochim Biophys Acta Gene Regul Mech.

[45] Inoue K, Tsuda M (2018). Microglia in neuropathic pain: cellular and molecular mechanisms and therapeutic potential. Nat Rev Neurosci.

[46] Guida F, de Gregorio D, Palazzo E, Ricciardi F, Boccella S, Belardo C, et al. Behavioral, biochemical and electrophysiological changes in spared nerve injury model of neuropathic pain. Int J Mol Sci [Internet]. 2020;21(9). Available from: https://www.mdpi.com/1422-0067/21/9/3396. 10.3390/ijms21093396 PMC7246983 32403385

[47] Wang X, Shen X, Xu Y, Xu S, Xia F, Zhu B, et al. The etiological changes of acetylation in peripheral nerve injury–induced neuropathic hypersensitivity. Mol Pain. 2018;14. 10.1177/1744806918798408 PMC6144590 30105933

[48] Fiskus W, Sharma S, Qi J, Valenta JA, Schaub LJ, Shah B (2014). Highly active combination of BRD4 antagonist and histone deacetylase inhibitor against human acute myelogenous leukemia cells. Mol Cancer Ther.

[49] Heinemann A, Cullinane C, De Paoli-Iseppi R, Wilmott JS, Gunatilake D, Madore J (2015). Combining BET and HDAC inhibitors synergistically induces apoptosis of melanoma and suppresses AKT and YAP signaling. Oncotarget.

[50] Zhao L, Okhovat JP, Hong EK, Kim YH, Wood GS (2019). Preclinical studies support combined inhibition of BET family proteins and histone deacetylases as epigenetic therapy for cutaneous T-cell lymphoma. Neoplasia (United States).

[51] Borgonetti V, Galeotti N (2021). Combined inhibition of histone deacetylases and BET family proteins as epigenetic therapy for nerve injury-induced neuropathic pain. Pharmacol Res.

[52] Doroshow DB, Eder JP, LoRusso PM (2017). BET inhibitors: a novel epigenetic approach. Ann Oncol.

[53] Proschak E, Stark H, Merk D (2019). Polypharmacology by design: a medicinal chemist’s perspective on multitargeting compounds. J Med Chem.

[54] Borgonetti V, Governa P, Biagi M, Pellati F, Galeotti N (2020). Zingiber officinale Roscoe rhizome extract alleviates neuropathic pain by inhibiting neuroinflammation in mice. Phytomedicine.

[55] Faivre EJ, McDaniel KF, Albert DH, Mantena SR, Plotnik JP, Wilcox D (2020). Selective inhibition of the BD2 bromodomain of BET proteins in prostate cancer. Nature.

[56] Gilan O, Rioja I, Knezevic K, Bell MJ, Yeung MM, Harker NR (2020). Selective targeting of BD1 and BD2 of the BET proteins in cancer and immunoinflammation. Science.

[57] Yang QQ, Zhou JW (2019). Neuroinflammation in the central nervous system: symphony of glial cells. Glia.

[58] Sommer C, Leinders M, Üçeyler N (2018). Inflammation in the pathophysiology of neuropathic pain. Pain.

[59] Subhramanyam CS, Wang C, Hu Q, Dheen ST (2019). Microglia-mediated neuroinflammation in neurodegenerative diseases. Semin Cell Dev Biol.

[60] Bromet E, Andrade LH, Hwang I, Sampson NA, Alonso J, de Girolamo G (2011). Cross-national epidemiology of DSM-IV major depressive episode. BMC Med.

[61] Kannan V, Brouwer N, Hanisch UK, Regen T, Eggen BJL, Boddeke HWGM (2013). Histone deacetylase inhibitors suppress immune activation in primary mouse microglia. J Neurosci Res.

[62] Patel AR, Patra F, Shah NP, Shukla D (2017). Biological control of mycotoxins by probiotic lactic acid bacteria. Dynamism dairy Ind Consum demands.

[63] Patnala R, Arumugam TV, Gupta N, Dheen ST (2017). HDAC inhibitor sodium butyrate-mediated epigenetic regulation enhances neuroprotective function of microglia during ischemic stroke. Mol Neurobiol.

[64] Suh HS, Choi S, Khattar P, Choi N, Lee SC (2010). Histone deacetylase inhibitors suppress the expression of inflammatory and innate immune response genes in human microglia and astrocytes. J Neuroimmune Pharmacol.

[65] Kaminska B, Mota M, Pizzi M (2016). Signal transduction and epigenetic mechanisms in the control of microglia activation during neuroinflammation. Biochim Biophys Acta Mol Basis Dis.

[66] DeMars KM, Yang C, Castro-Rivera CI, Candelario-Jalil E (2018). Selective degradation of BET proteins with dBET1, a proteolysis-targeting chimera, potently reduces pro-inflammatory responses in lipopolysaccharide-activated microglia. Biochem Biophys Res Commun.

[67] Baek M, Yoo E, Choi HI, An GY, Chai JC, Lee YS (2021). The BET inhibitor attenuates the inflammatory response and cell migration in human microglial HMC3 cell line. Sci Rep.

[68] Wang H, Huang W, Liang M, Shi Y, Zhang C, Li Q (2018). (+)-JQ1 attenuated LPS-induced microglial inflammation via MAPK/NFκB signaling. Cell Biosci.

[69] Hajmirza A, Emadali A, Gauthier A, Casasnovas O, Gressin R, Callanan MB (2018). BET family protein BRD4: an emerging actor in NFκB signaling in inflammation and cancer. Biomedicines..

[70] Guo S, Perets N, Betzer O, Ben-Shaul S, Sheinin A, Michaelevski I (2019). Intranasal delivery of mesenchymal stem cell derived exosomes loaded with phosphatase and tensin homolog siRNA repairs complete spinal cord injury. ACS Nano.

[71] Borgonetti V, Galeotti N (2021). Intranasal delivery of an antisense oligonucleotide to the RNA-binding protein HuR relieves nerve injury-induced neuropathic pain. Pain.

