# Supplementary material for: Dual HDAC/BRD4 Inhibitors Relieves Neuropathic Pain by Attenuating Inflammatory Response in Microglia After Spared Nerve Injury
Source: Neurotherapeutics. 2022 May 2;19(5):1634–48. doi: 10.1007/s13311-022-01243-6 (PMC9606187; doi:10.1007/s13311-022-01243-6)
Supplement: Supplementary file 1 — Supplementary file1 (PDF 227 kb). Supplementary Figure S1. Time course of the effect on acute pain of SUM35 (A; 10, 50, 100, 250 and 500 µM) and SUM52 (B 10, 50, 100, 250 and 500 µM) at 30, 60, 90, 120 and 180 min, using the Hot Plate Test in naïve mice. n=8 for each group. C) Experimental protocol of SUM35 and SUM52 intranasal administration. Time course of the effect on persistent pain of SUM35 (D; 10, 50, 100, 250 and 500 µM) and SUM52 (E; 10, 50, 100, 250 and 500 µM) at 30, 60, 90, 120 and 180 min, using the Plantar Test in SNI mice. n=8 for each group. F) Experimental protocol used for SNI mice. Time course of the effect on persistent pain of SUM35 (G; 10, 50, 100, 250 and 500 µM) and SUM52 (H; 10, 50, 100, 250 and 500 µM) at 30, 60, 90, 120 and 180 min, using the Von Frey Test. n=8 for each group [file 13311_2022_1243_MOESM1_ESM.pdf]

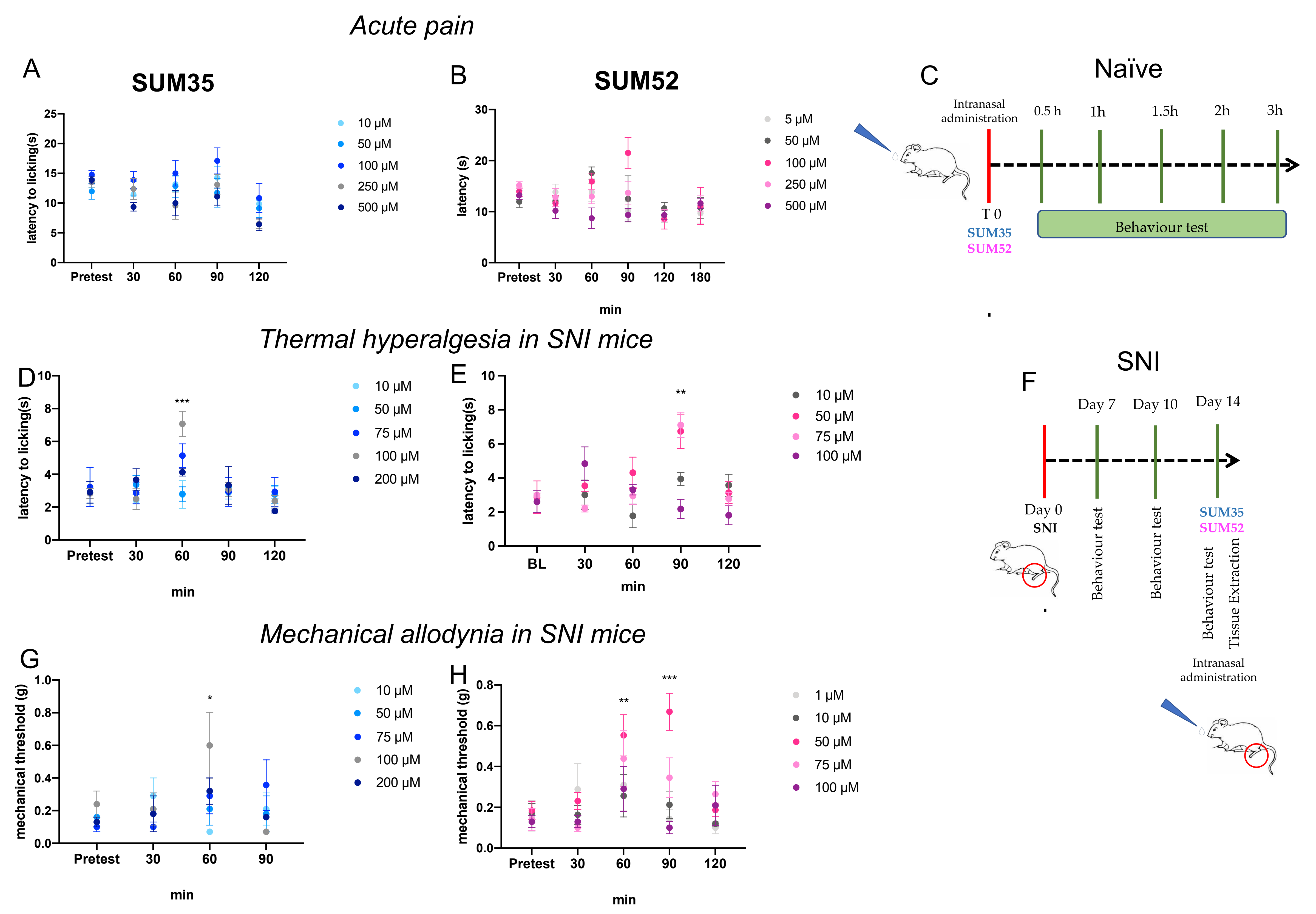

Acute pain
A
SUM35
latency to licking(s)
Pretest
30
60
90
120
10 µM
50 µM
100 µM
250 µM
500 µM
B
SUM52
latency (s)
Pretest
30
60
90
120
180
min
5 µM
50 µM
100 µM
250 µM
500 µM
C
Naïve
Intranasal administration
0.5 h
1h
1.5h
2h
3h
T 0
SUM35
SUM52
Behaviour test
Thermal hyperalgesia in SNI mice
D
latency to licking(s)
***
Pretest
30
60
90
120
min
10 µM
50 µM
75 µM
100 µM
200 µM
E
latency to licking(s)
**
BL
30
60
90
120
min
10 µM
50 µM
75 µM
100 µM
SNI
F
Day 7
Day 10
Day 14
Day 0
SNI
Behaviour test
Behaviour test
SUM35
SUM52
Behaviour test
Tissue Extraction
Intranasal administration
Mechanical allodynia in SNI mice
G
mechanical threshold (g)
*
Pretest
30
60
90
min
10 µM
50 µM
75 µM
100 µM
200 µM
H
mechanical threshold (g)
**
***
Pretest
30
60
90
120
min
1 µM
10 µM
50 µM
75 µM
100 µM
